# A RGBD-Based Interactive System for Gaming-Driven Rehabilitation of Upper Limbs †

**DOI:** 10.3390/s19163478

**Published:** 2019-08-09

**Authors:** Gabriel Fuertes Muñoz, Ramón A. Mollineda, Jesús Gallardo Casero, Filiberto Pla

**Affiliations:** 1 Edison Desarrollos, S.L., 44002 Teruel, Spain; 2 Institute of New Imaging Technologies, Universitat Jaume I, 12071 Castellón, Spain; 3 E.U Politécnica, Universidad de Zaragoza, 44003 Teruel, Spain

**Keywords:** rehabilitation, upper limbs, kinect, augmented reality, usability study, RGBD sensor

## Abstract

Current physiotherapy services may not be effective or suitable for certain patients due to lack of motivation, poor adherence to exercises, insufficient supervision and feedback or, in the worst case, refusal to continue with the rehabilitation plan. This paper introduces a novel approach for rehabilitation of upper limbs through KineActiv, a platform based on Microsoft Kinect v2 and developed in Unity Engine. KineActiv proposes exergames to encourage patients to perform rehabilitation exercises prescribed by a specialist, controls the patient′s performance, and corrects execution errors on the fly. KineActiv comprises a web platform where the physiotherapist can review session results, monitor patient health, and adjust rehabilitation routines. We recruited 10 patients for assessing the system usability as well as the system performance. Results show that KineActiv is a usable, enjoyable and reliable system, that does not cause any negative feelings.

## 1. Introduction

Exercises that are prescribed, regulated and controlled, help people with motor difficulties to recover mobility in the affected limbs and, therefore, quality of life. A frequent practice is to have specialized personnel in the rehab sessions (i.e., physiotherapists), who supervise the quantity and quality of the exercises. An alternative to specialized personnel is the use of robots designed to assist patients in performing rehabilitation exercises [1], which is usually possible only in specialized or clinical environments (e.g., hospitals). However, these are expensive solutions and may imply the displacement of patients with reduced mobility.

A cheaper but less effective approach is to perform the exercises prescribed independently at home, without the supervision of a professional. However, a study indicates that only 31% of the people that suffer from a mobility disorder execute the exercises correctly [2]. In addition, loss of patient motivation is frequent.

The proliferation of increasingly cheaper sensors has allowed for the creation and development of systems capable of automatically controlling the frequency, duration and correction of the prescribed exercises [3,4,5]. In addition, the use of multimodal user interfaces, together with the gaming technologies, could help to keep the user motivated and engaged during the execution of the exercises. The ultimate goal is to maintain patient adherence to treatment, which is particularly important in long-term rehabilitation due to the need to reduce recovery periods [6].

This work introduces KineActiv, a system designed to replace physiotherapists in supervising upper limb exercises, helping the patient to achieve a more effective and faster rehabilitation. The system relies on an RGBD (Red, Green, Blue, Depth) sensor (MS-Kinect V2) and on a friendly, interactive, gaming-based user interface, aimed at making the execution of the exercises easier and enjoyable. The system quantitatively measures upper-limb movements and compares them with expected goals previously established by the physiotherapist. It also allows the specialist to perform real-time monitoring of the progress of the patients through a web-based system, with statistics that summarize patients′ performance.

Experiments were conducted on a real dataset consisting of quantitative measurements obtained from 10 patients, when they performed a number of sessions of a prescribed rehabilitation routine. Statistical analysis allowed us to evaluate the accuracy and reliability of the system, as well as its sensitivity at measuring the progress of patients.

This manuscript is an extended version of a previous conference paper [7], which was limited to a usability study on the interactive user interface of KineActiv aimed at measuring the patient′s immersion experience. Unlike, Chang, Y.J. [4], this paper provides a comprehensive evaluation of KineActiv, bringing together a quantitative analysis of its effectiveness as a rehabilitation tool, a detailed description of its operational workflow, and the aforementioned usability study. The effectiveness study is intended to establish the accuracy and sensitivity of KineActiv, while the workflow description stresses the opportunities for real-time monitoring of the degree of correction of prescribed exercises. Finally, this work includes a much broader review of the state of the art development.

## 2. Related Work

The performance of Microsoft Kinect as a tool to evaluate kinetic variables, as compared to more conventional solutions, is a subject of great interest [5]. Chang [4] compared Kinect against the high-fidelity OptiTrack system, showing that the former can achieve competitive motion tracking performance. Kinect robustness at modelling the human skeleton in the presence of partial occlusions has promoted its use as a marker-free motion monitoring system, being a low-cost solution as regards more specialized vision-based technologies (BTS, Vicon, OptiTrack, etc.). Naeimabadi et al. [8] evaluated the accuracy and usability of the Kinect 1.0, 2.0 and wearable devices for tele-rehabilitation of the knee. A routine of exercises was defined to measure the angle of the knee and determines the accuracy. Their findings showed that the second generation of Kinect and wearable sensors have acceptable accuracy, with average errors of 2.09°, 3.11° and 4.93° for Kinect 2.0, accelerometers and inertial measurement units, respectively. Tanaka et al. [9] conducted a study aimed at assessing the accuracy of Kinect compared to a marker-based motion capture system. They concluded that, in spite of differences with marker-based systems, Kinect could be useful for accurately classifying movements. Zhou and Hu [10] conducted a review of movement systems for rehabilitation. Six criteria were considered: cost, size, weight, function, operation and automation. Marking-free visual systems were stressed because of their small dimensions, robust performance and low cost.

Other studies have shown that Kinect is able to measure gross movements, which makes it suitable for stroke rehabilitation, Napoli et al. [11] or for measuring motion disorders in people with Parkinson′s disease [12,13]. Varona, J. et al. [14] and Zanatta, J. et al [15] study, a Kinect-based system with an interactive virtual environment was successfully used for rehabilitation of upper limbs in stroke patients. Based on motion data and signals obtained from ergonomic measuring devices, the system monitors and assesses the rehabilitation progress. It has also been determined that Kinect device can track body movement with the precision required for standard equilibrium tests [16], such as foot balance assessment [17]. Kinect sensitivity was also stated in, Obdrzalek, S. et al. [18], where postures of elderly people in standing and sitting positions were accurately estimated. Results proved to be very useful in active therapy exercises.

Rehabilitation of brain injuries has also been addressed through virtual reality [19]. Da Costa and de Carvalho [20] and Edmans [21] showed the positive results of a virtual reality device for cognitive rehabilitation. The potential of virtual reality in stroke patients was investigated by Rand [3] and Broëren [22]. Following these results, Pyk et al. [23] presented a virtual reality therapy system for the rehabilitation of arms and hands in children. They verified that the system could reduce the therapy cost, increase patient motivation and objectively evaluate the progress made. Several studies have shown that by offering virtual rehabilitation exercises designed as games, it is possible to motivate patients to perform rehabilitation exercises, and also to increase their adherence to treatment, as we can see in, Lange, B., Flynn, S., Lozano, J. et al. [24,25,26].

Examples of integration of Kinect, gaming and virtual reality for motor rehabilitation purposes of patients with brain injuries can be found in, Pyk, P., Cabrera, R., Jung, I., Jonsdottir, J. et al. [23,27,28,29]. They have been designed to simply encourage patients to do exercises, without keeping a precise control of the movement. That is, they are intended to educate the brain to recover a lost function, or to mitigate movement degeneration, by roughly requiring a patient to reach a goal.

Unlike the vast majority of previous works, which focus on neurorehabilitation, this paper concerns the use of a Kinect device to accurately measure kinematic parameters of upper limbs, within a gamified and augmented environment. There exist commercial systems with some similarities to the one described here. Kinovea [30] is a software of sports biomechanics, which is also used in physical rehabilitation. It is a video analyzer that evaluates, corrects and keeps track of movements. It measures times, angles, trajectories, perspectives and coordinates. Despite being a powerful software, the movement must be recorded and then studied. Skill Spector [31] also records videos first to perform later offline analyses. The system requires the user to manually locate the joints, from which it generates a model for the analysis. Another Kinect-based example is Diaple [32], in which the patient must imitate video-recorded exercises done by physiotherapists. Then, the system measures how similar the patient′s movements are to those of the physiotherapist. This similarity score can be considered as general feedback, that is to say, no visual cues are given to the patient to correct their personal execution in real time. Other commercial systems are based on sophisticated multi-camera systems that record patients wearing special suits within a controlled scenario. However, they are generally costly and require calibration tasks, thus their use is usually constrained to specialized environments.

## 3. Materials and Methods

### 3.1. System Overview

**Figure 1:**
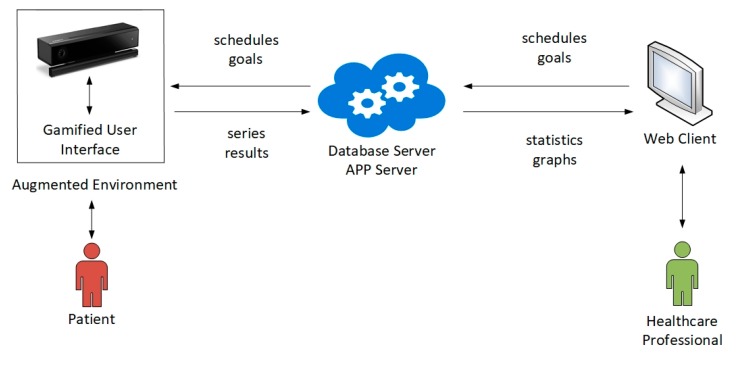
System Architecture of KineActiv.

KineActiv is intended to assist physiotherapists in the recovery phase of patients with upper limb disorders. Patients interact with a gamified user interface, which implements a customized game environment for each type of exercise. Motions of the patient′s limbs are automatically tracked, measured and assessed against a number of goals set in advance by the medical personnel, allowing the system to provide real-time feedback on the correction of the exercise. Besides, the system records exercise data and makes them available to the physiotherapist through a client-server architecture, so that future sessions can be planned accordingly. Figure 1 illustrates the system architecture.

The operational workflow of KineActiv can be outlined as follows:

1. **Calibration.** The system captures the patient′s biometric data, and adapts exercises to each patient.
2. **Execution and control.** Depending on the prescribed exercise, the system presents a customized game environment to the patient, where goals are represented through computer-generated visual elements. The patient is encouraged to perform the corresponding exercise. The system checks whether the execution meets the goal, according to configurable tolerance margins. The system provides real-time visual feedback to the patient, indicating to what extent the goal is reached. When the patient does not perform the exercise in a correct way, the system notifies the patient, and allows the patient to continue the series of exercises.
3. **Data registration.** When the execution of an exercise ends, all the series of results are stored in a database located on a server.
4. **Exercise analysis and scheduling.** Healthcare professionals can access their private web areas, where statistics and charts provide details on the execution of the exercises, emphasizing the distance to the goal in each case. After judging results, new sessions can then be scheduled.

Next section provides insight into the theory and methods behind each system stage.

### 3.2. System Stages

#### 3.2.1. Acquisition

The acquisition stage consists of gamified user environments developed in the Unity game engine, which integrate services provided by the Kinect SDK 1.8 through the Unity package Kinect with MS-SDK. Since the goal of this work is to treat shoulder injuries, only the 3D coordinates of the shoulder, elbow and the wrist (joints of interest) are obtained and tracked in real time. These coordinates are high-precision data calculated 30 times per second, so updated joint locations are available whenever they are required.

Before starting any exercise, the system checks whether the patient is correctly positioned and in upright posture. The patient is expected to be at a distance of 2 to 4 m from the Kinect sensor, standing up straight in a position of the scene determined as optimal by the system considering the injured arm. Although the Kinect device can work reliably from 0.4 to 8.5 m, it is well known that the optimal working distance range is from 1.4 to 4.5 m [33].

**Figure 2:**
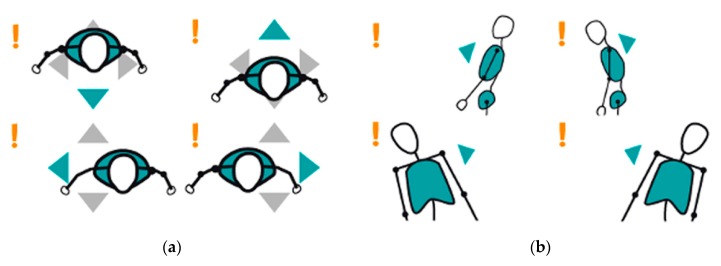
The system indicates the patient how to correct the position in the scene (**a**) and the body posture (**b**).

**Figure 3:**
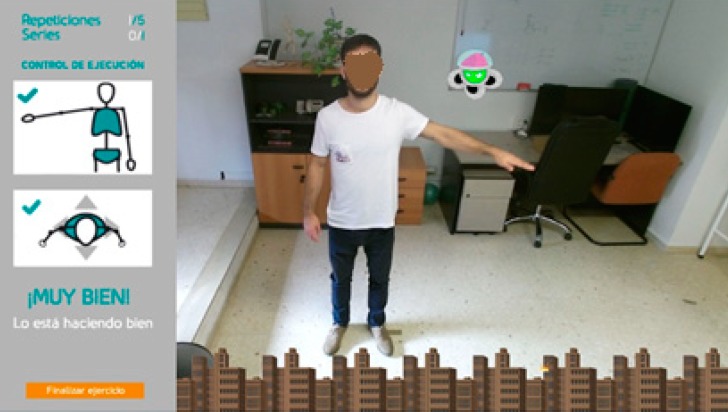
Gamified environment designed to encourage concentric exercises.

The system is capable of detecting incorrect positions and body postures, and of indicating in which direction the patient must move to meet the acquisition requirements. These instructions are conveyed through visual elements located in a panel placed either to the right or to the left of the main scene, depending on the arm that requires rehabilitation. Figure 2 illustrates examples of different visual instructions to correct the patient′s position (Figure 2a, zenithal view) and the body posture Figure 2b. Only when all the requirements are met, the system allows to start the exercise. A full view of the acquisition interface, including these controls, can be found in Figure 3.

#### 3.2.2. Gamified User Environments

This study focuses on two types of exercises: concentric and isometric. The first one consists of repeatedly elevating the arm until reaching an objective angle, as established by the physiotherapist. In the second type, the arm is raised until reaching an objective angle, and keeps that position for a certain period of time determined by the specialist. In both cases, goals are personalized, depending on the circumstances of each patient.

To encourage patients to achieve their goals, specific gamified environments have been designed and implemented for each type of exercise.

The execution of the concentric exercises is part of a game in which a computer-generated alien, located in the exact place to which the patient must raise his arm (goal), is willing to destroy a city if the patient fails to achieve the established goals. Figure 3 illustrates this environment. The city is represented by a skyline on the lower edge of the image, thus avoiding to affect the real-world scene. The alien is designed to follow the patient in case the patient moves during the execution of the exercise, so that it always keeps the target position.

Two main scenarios can be identified in this exergame:

- **Positive.** The patient performs a correct execution of the exercise, reaching the alien, so destroying it and keeping the city safe. Once the patient lowers the arm, another alien is generated at the same target position.
- **Negative.** The patient performs the exercise incorrectly, and fails to reach the objective position. Then, the alien attacks the city with fireballs.

The system records how many successful repetitions and how many failures have been made in each series, and stores the information in the cloud database.

The isometric exercise takes advantage of a game that is very different from the previous one. It consists of a bird flying over the patient′s hand and an empty cage located in the exact place (target degree) where the patient must keep his hand for a certain period of time (goal). The patient is expected to get the bird back into the cage. This game can also be summarized in two scenarios:

- **Positive.** The patient performs a correct execution of the exercise, reaching the cage and keeping the bird in the cage during the prescribed period of time on a continuous basis, so the bird alights and rests.
- **Negative.** The patient performs the exercise incorrectly, failing to keep the bird in the cage during the prescribed period of time on a continuous basis, so the bird continues flying.

The system records what fraction of the expected time the patient was able to keep the bird in the cage at each repetition, and stores this information in the cloud database.

The suitability of exergames for rehabilitation has been studied considering factors such as facilitators and barriers [34], where “perceived ease of use of the exergames” and “possibility of providing additional therapy” were identified as the main facilitators at the technological level. In the case of KineActiv, the perceived ease of use has been verified by the usability study in Section 4.2, whereas additional therapy can be attained as medical personnel can control the performance of the patients and adapt the prescribed exercises on the fly. With regard to barriers, [34] identified the low precision in motion capture as the main discouraging factor. However, as explained in Section 4.3, the KineActiv accuracy and reliability when measuring patients′ movements have also been clearly established. Summarizing, this work successfully addresses typical facilitators and barriers of exergames for rehabilitation purposes.

#### 3.2.3. Monitoring and Measurement

The monitoring and measurement stage can be divided into the following tasks:

1. Patient-based calibration
2. Tolerance margin setup
3. Joint monitoring
4. Occlusion handling

##### Patient-Based Calibration

Given a patient, the first step is intended to capture their biometric data, so that limits associated to prescribed exercises can be reliably determined. It ensures that the patient performs the exercises within a customized environment.

Once the patient is well positioned and upright, the 3D coordinates of the shoulder, elbow and wrist are used to compute the arm length (distance from the shoulder to the wrist). An Euclidean vector is then defined taking the shoulder coordinate as the initial point, the arm length as its magnitude, and using the type of action (abduction, flexion) and the angle established by the physiotherapist to define the vector direction. The end point of this vector determines the personalized target position of the exercise, where the gamified elements will be generated.

##### Tolerance Margin Setup

The tolerance margins define a customized 3D region for each patient and exercise, outside of which any movement is considered an incorrect execution. It is also considered a calibration step, because margins depend on the patient´s condition.

**Figure 4:**
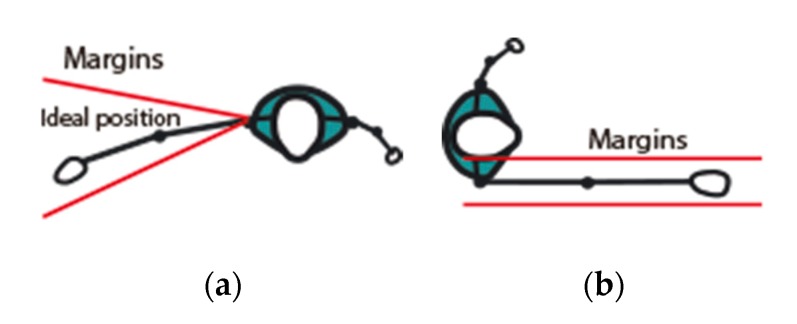
Tolerance margins for abduction (**a**) and flexion (**b**).

Two types of motions have been considered in this work: abduction and flexion. They start from a standing position, with arms parallel to the trunk. In abduction, the arm of interest is raised straight in the “plane of the trunk” up to reach the target angle. Flexion is similar, except that the arm is raised to the front, in a plane perpendicular to that of the trunk. In both actions, the target position of the hand at the end of the motion is modelled by a gamified element. Figure 4 illustrates the tolerance margins established for abduction and flexion (zenithal view).

In abduction exercises, two tolerance margins (front and rear) are defined from two non-parallel planes whose intersection is determined by the coordinates of the shoulder Figure 4a. This open geometry admits abduction exercises of patients with a wide spectrum of physical conditions. In flexion, the physiotherapist establishes two parallel margins of tolerance, which are, in turn, perpendicular to the plane of the trunk Figure 4b. In each case, the two margins delimit a valid 3D region, which cannot be exceeded by any joint at any time. When a joint breaks a margin, the system interprets the error, and informs the patient how to correct the exercise. For example, if the system detects an elbow outside the margins (the patient is bending the arm), the system asks the patient to fully extend the arm. For the session records, the repetition is interrupted and the angle reached at the time of the interruption is annotated. These margins remain virtually attached to the patient, so if the patient moves (within acceptable limits), margins are updated according to the new joint coordinates.

##### Joint Monitoring

Kinect provides high-precision joint coordinates 30 times per second. The precision of each coordinate value is represented by more than 20 decimal places; the mere fact of breathing causes the positions of the joints to change. Fortunately, such high precision is not necessary at all to monitor rehabilitation exercises, so a lower precision of three decimal places was adopted after proving to be sufficient for detecting significant changes. In this way, the complexity of data and software was reduced without losing effectiveness.

##### Occlusion Handling

Abduction exercises are very easy to monitor, because all the arm joints remain visible throughout the execution time. On the contrary, flexion might entail joint occlusion or loss of tracking for a small range of angles, where the joints are aligned with the sensor. In these cases, Kinect provides estimates of the positions of the occluded joints. By the time the joint becomes visible again we can ensure its position. Dealing with this situation is simple and effective. If the actual positions of the occluded joint just before and after the occlusion were correct, then it can be fairly assumed that the joint kept correct positions while it was occluded. Otherwise, in case the joint had broken the alignment, it would have been detected by the sensor again, and its actual position assessed with respect to the tolerance margins.

#### 3.2.4. Website

KineActiv includes a website responsible for managing medical appointments, registering patients, scheduling rehabilitation sessions, and assessing results and patients′ evolution. Physiotherapists can access their private section, where the following functional scopes can be found:

1. To prescribe an exercise routine.
2. To perform a system calibration.
3. To assess a patient´s evolution.

Physiotherapists can prescribe rehabilitation routines of exercises implemented in KineActiv. These exercises are customizable through a set of predefined characteristics, and are linked to the treatment of specific joints. Therefore, physiotherapists can select and configure them according to the patient′s needs, for instance, by pre-setting the number of sessions, target degrees, number of series, repetitions per series, execution time, etc. The clinical staff can also send the patient a report with the results of the rehabilitation process.

Physiotherapists can also calibrate the system for each patient whenever they consider it necessary to adapt the exercises to the patient′s progress and condition.

With regards to the assessment of patient′s progress, KineActiv generates reports at patient level, with graphic and statistics of multiple sessions, reports at the session level, with graphic and statistics of multiple series, and reports at the level of specific series, with results separated by repetitions.

**Figure 5:**
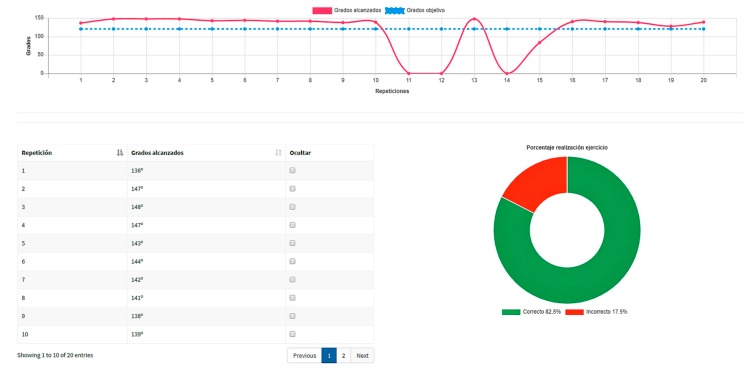
A report of a series of 20 repetitions of a concentric exercise.

Figure 5 shows a report of a series of 20 repetitions of a concentric exercise, which consists of a line chart displaying the performance (degrees achieved) by individual repetitions (pink curve), together with the established goal (blue line), a table comprising the exact figures of repetition results, and a doughnut chart summarizing the percentages of correct repetitions in green (the goal is achieved) and of incorrect repetitions in red (the goal is not achieved).

**Figure 6:**
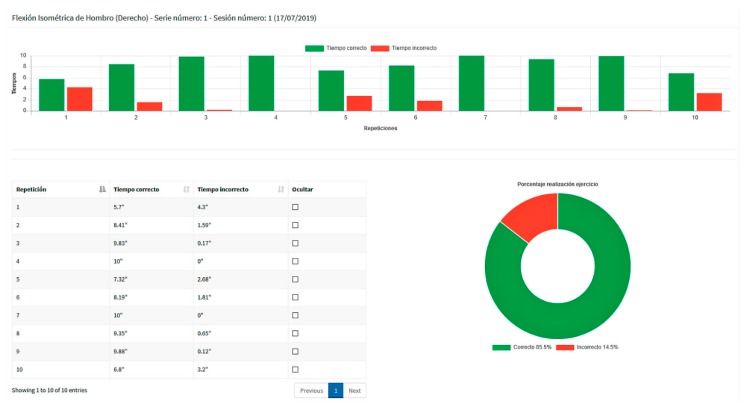
A report of a series of 10 repetitions of an isometric exercise.

In the case of reports of individual series of isometric exercises, Figure 6 illustrates an example of a series of 10 repetitions composed of a column chart showing, for each repetition, the correct and the incorrect execution times in the green and red columns, respectively, a table comprising the exact figures, and a doughnut chart summarizing the percentages of accumulated times of correct and incorrect execution.

In the design of the web user interface, some well-known usability heuristics [35] were considered:

- **Visibility of system status.** Reports reflect real-time results, which are updated as soon as the execution of the exercises ends.
- **User´s preferred language.** The terminology matches the specialized language used by medical personnel, approaching the system to the real world.
- **Consistency with expectations.** Graphics uses the green color to identify correct exercises and the red one to represent incorrect exercises.

#### 3.2.5. Web Server

The communication between the KineActiv environment for acquisition and control of patients′ motions and the website of the clinical area is established by a cloud solution provided by Amazon Web Services. It includes a database with structured information about patients, medical personnel, exercises, calibration data, pathologies, guidelines, series results, etc. All this information is either produced or consumed by the patient′s environment or the medical staff′s website.

## 4. Results

Two studies were conducted to evaluate KineActiv. Firstly, a usability study was performed with the enrolled patients by means of a questionnaire Section 4.2. Secondly, a performance study was carried out to empirically prove the system accuracy and the system sensitivity at assessing upper-limb disorders Section 4.3.

Samples were recorded using a MS Kinect device under a spatial resolution of 1920 × 1080 pixels, a mean distance to sensor of 2.5 m, a horizontal field of view of 70 degrees, a vertical field of view of 60 degrees, and a frame rate of 30 frames/sec.

### 4.1. Participants

Ten adult patients with some diagnosed arm injury were involved in experiments, distributed into six males and four females with ages ranging from 38 to 83 years old. They were recruited in the SEAP Polyclinics (http://www.policlinicasseap.com/centros/policlinica-teruel/), a collaborating center that is evaluating KineActiv in its Physiotherapy service. Data consists of series of quantitative measurements obtained from each patient, when performing four upper-limb exercises. Eligible patients were those who showed a clear pattern of health improvement throughout the sessions, which was important to assess the measurements provided by the system when dealing with different health conditions. Data were also acquired from a professional physiotherapist doing the same exercises prescribed to patients, to help establish the accuracy of the system and to be used as control data. Participants signed a written consent form, agreeing to take part in the study subject to their personal data remain confidential. Table 1 shows the patients′ profiles.

Recording took place in a research laboratory where background and illumination were constant. Patients were instructed about the exercise layout (3 weeks × 2 weekly sessions × 4 exercises × 3 series), and they were asked to let themselves be guided through the user interface. The four exercises are combinations of the types of upper-limb motion (abduction, flexion) and the two dynamics (isometric, concentric). Each exercise led to a particular measure.

### 4.2. Usability Study

After analyzing questionnaires used for similar purposes in the field of rehabilitation and virtual health [36,37], our usability questionnaire was designed by combining the items present in two questionnaires that match the goals pursued in this study. Firstly, we adopted the ten items defined in the SUS (System Usability Scale) questionnaire [38], that have been used in the usability assessment of a rehabilitation system for the upper limbs [39]. Besides, in order to gather more information, we added the six items used in, Shin, J. et al. [40] for evaluating flow in a virtual reality rehabilitation system, adopted from, Park, J. et al. [41]. These items were taken into account since the gamification approach is expected to cause a full immersion in the system, guiding a patient to achieve the goals set out by the physiotherapist. In this way, we have been able to test both usability and flow, when playing with the games included in our system. A total of 16 items were put together in one questionnaire, where every item needs to be scored between 1 and 5, with 1 and 5 being the lowest and the highest values, respectively. Table 2 shows the average values and standard deviations of all items. The items from 1 to 10 are those from the SUS questionnaire, while items from 11 to 16 are those corresponding to flow. Based on the results of the first ten items, the SUS score of the system, a usability measure ranging from 0 to 100, was computed. The overall result was 84.5, a value that establishes the suitability of KineActiv as a usable system.

The second part of the questionnaire, as we have already stated, is about flow when using the system. The flow items (11–16) evaluate three different factors. The first one is attentional focus (items 11 and 12). In this case, the values obtained were not the ones desired, which suggests that the game may not maintain the attention in a strong manner. The second factor is intrinsic interest or pleasure (items 13 and 14). In this case, the values received by the items are good (1.6 in a question where boredom was valued and 4.4 in the opposite one, where they were asked by fun). Thus, the system was considered enjoyable. Lastly, the control was also evaluated (items 15 and 16). Here, values were also good, showing that the use of KineActiv did not cause any negative feelings.

According to all above discussion, the validation of KineActiv usability yielded satisfactory results. The system proved to be easy to use and the flow experience was considered interesting and fun. Also, considering the results of this study, we can state that the gamified activities included in the system attract users and catch their attention. This can be concluded as the items about attentional focus and intrinsic interest received acceptable values in the study.

### 4.3. Performance Study

This study is intended to establish the accuracy and the sensitivity of KineActiv, by examining the distributions of four measures over patients as a function of the rehabilitation session. As referred above, these measures were defined to quantify the progress of patients in goal achievement when performing the four upper-limb exercises. These four measures were formulated as follows:

**Isometric-based measures**. Two measures were defined from the isometric evaluation of both types of motion: The Isometric Abduction Index (IAI) and the Isometric Flexion Index (IFI). In each case, the participant was asked to keep the hand within the computer-generated 3D region (the cage) for 45 s, while the system provided real-time visual feedback on exercise correction and execution time. The amount of seconds in which this goal was verified was given as the measure value.

**Concentric-based measures**. Two measures were defined from the concentric evaluation of the two motions: The Concentric Abduction Index (CAI) and the Concentric Flexion Index (CFI). The participant was asked to repeat the corresponding exercise 20 times, and the angle of the arm with respect to the body was measured in each repetition.

Summarizing, each patient′s data is composed of the following pieces:

**IAI** Six daily sessions of three series of a single isometric abduction each, resulting in 18 IAI measurements in total.

**IFI** Six daily sessions of three series of a single isometric flexion each, resulting in 18 IFI measurements in total.

**CAI** Six daily sessions of three series of 20 concentric abductions each, resulting in 360 CAI measurements in total.

**CFI** Six daily sessions of three series of 20 concentric flexions each, resulting in 360 CFI measurements in total.

#### 4.3.1. System Accuracy

This section includes two analyses aiming at providing evidence in favor of the accuracy of KineActiv. The first one involves a healthy physiotherapist, who was asked to perform an abduction and a flexion up to 80°, as established by a goniometer. Both exercises were also measured by the Kinect-based system, yielding 80.12° and 80.06° in abduction and flexion, respectively. That is, the relative errors of the KineActiv with respect to the goniometer were 0.15% and 0.075% in each case. The physiotherapist also carried out the same exercises done by the patients, carefully following the instructions. Mean results, measured by the system, were IAI = 43.65 s, IFI = 44.05 s, CAI = 120° and CFI = 120°. That is, measurements were very close to expectations, which can be considered a successful first performance test on the system.

The second perspective of analysis is based on the distribution of standard deviations of CAI and CFI across patients, as functions of the rehabilitation session. These measures were chosen due to their numerous repetitions, which makes them more appropriate for descriptive statistics.

The rationale of using distributions of standard deviations is that narrow distributions of small deviations would empirically demonstrate the stability of the system, while measuring different executions of the same action. Note that each single measurement is the result of a particular execution of a given action, unique by nature, and the system acquisition error. Thus, small deviations from the average can reasonably be assumed to reflect a reliable system.

**Figure 7:**
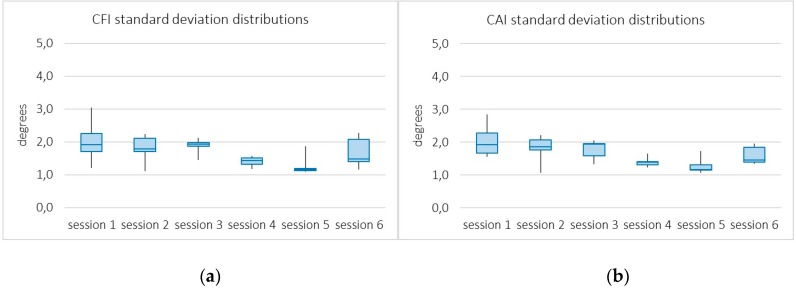
Box plots from distributions of the standard deviations of the Concentric Abduction Index (**a**) and the Concentric Flexion Index (**b**) across patients over the six sessions.

Figure 7 shows box plots representing the distributions of standard deviations across patients for each measure and daily session. As observed, they are very tight distributions of small deviations, most of them between 1 and 2 degrees in angle measurements in the range 80–120 degrees. This level of disagreement is consistent with the Kinect error of 2.09 degrees reported by Naeemabadi, M. et al. [8]. Deviations tend to decrease with the progress of rehabilitation (with performance improvement), with the exception of the last session, possibly the most demanding one. This second analysis also shows the very reliable behavior of the system.

#### 4.3.2. System Sensitivity

**Figure 8:**
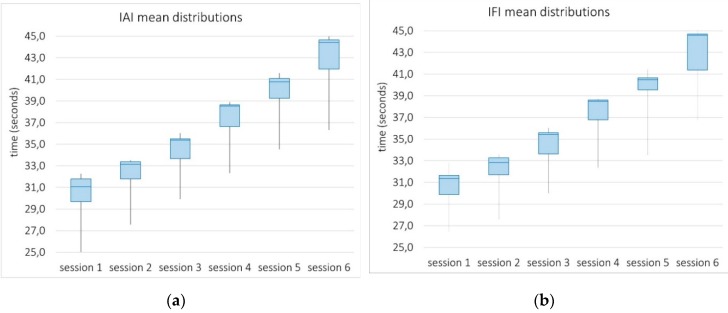
Box plots from distributions of the mean values of the Isometric Abduction Index (**a**) and the Isometric Flexion Index (**b**) across patients over the six sessions. These measures ranges from 0 to 45 s, with the latter being the goal established in this study.

Figure 8 depicts the distributions of mean values of patients′ isometric-based measurements as a function of the session. The domain of these measures ranges from 0 to 45 s, with the latter being the goal established by the physiotherapist in this study. Thus, the higher the value, the better the execution. Resulting box plots can be considered narrow, except for the lower whisker, suggesting a high level of agreement among the majority of patients. In particular, the lower whisker is stretched by patient number eight, who performed considerably below the rest. When looking more closely at results, most distributions (box plots) are strongly left-skewed. This means that the half of patients with poorer performance (below the median) progress more unequally, while those patients who are closest to goals, progress more evenly.

**Figure 9:**
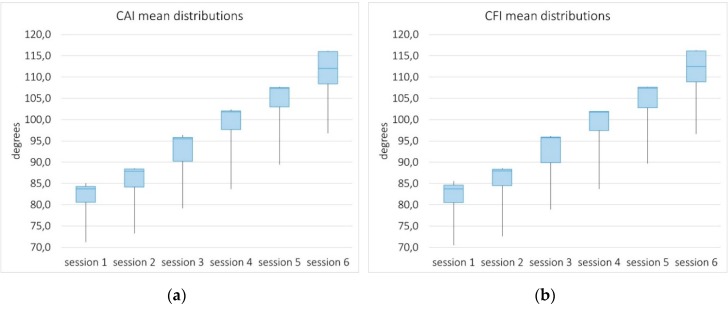
Box plots from distributions of the mean values of the Concentric Abduction Index (**a**) and the Concentric Flexion Index (**b**) across patients over the six sessions. These measures ranges from 0 to 120°, with the latter being the goal established in this study.

This result is somewhat consistent with intuition: there is more dispersion among patients with greater disorders. Figure 9 shows similar distribution patterns of the two concentric indexes. The domain of these indexes ranges from 0 to 120°, with the latter being the goal established by the physiotherapist in this study. Thus, the higher the value, the better the execution.

## 5. Conclusions

This work has presented a RGBD-based interactive system (KineActiv) which proposes a holistic approach to the rehabilitation of upper limbs. KineActiv provides a gamified and augmented user interface designed to replace physiotherapists in the supervision of upper limb exercises. The system guides patients through customized games on augmented scenes, which aims at encouraging patients to achieve a number of goals established by the physiotherapist. KineActiv tracks and measures limb motions, compares them against goals, and provides real-time feedback to patients about whether the exercise is meeting the expected goals. Our approach includes a web platform that allows the specialist to monitor the progress of patients by means of statistics, tables and charts. The main goal has been to create interactive, simple to use and fun environments that favor more effective and faster rehabilitation processes, while keeping patients engaged.

Two studies were conducted to evaluate KineActiv based on the experience of ten patients. Firstly, a usability questionnaire was designed to measure both usability and flow when using the gamified environments. Secondly, a functionality study was performed to establish the accuracy and the sensitivity of KineActiv, by examining the distribution of four measures of limb exercises over patients as a function of the rehabilitation session. Results empirically prove that KineActiv is a usable, enjoyable and effective system, that does not cause any negative feeling.

Several issues remain for future research. The system is open to new exercises and associated games to rehabilitate any part of the body, as well as to improve the quality of games with better graphics and more engaging goals. For example, it would be interesting to conduct competitions among patients with similar injuries, in order to maintain adherence to treatment.

## Figures and Tables

**Table 1:** Patient´s profiles.

Patient Id	Gender	Age	Injury
1	male	42	joint dislocation
2	male	47	tendinopathy
3	female	64	humerus fracture
4	male	67	joint dislocation
5	male	38	tendinopathy
6	female	55	tendinopathy
7	female	57	calcification
8	male	83	osteoarthritis
9	male	45	shoulder impingement
10	female	50	shoulder impingement

**Table 2:** Results of the usability study [5].

Question	Average Value	Standard Deviation
1. I think I would like to use KineActiv frequently	4.7	0.48
2. I think that KineActiv is unnecessarily complex	1.4	0.52
3. I think that KineActiv is easy to use	4.5	0.53
4. I think that I would need help to use KineActiv	2.3	1.06
5. I think that the various functions in KineActiv are well integrated	4.3	0.67
6. I think there is too much inconsistency in KineActiv	1.4	0.52
7. I imagine that most people would learn to use KineActiv very quickly	4.6	0.52
8. I found KineActiv very cumbersome to use	1.5	0.53
9. I felt very confident using KineActiv	4.4	0.52
10. I would have needed to learn a lot of things before using KineActiv	2.1	0.88
11. I thought about other things when using KineActiv	2.5	0.85
12. I was aware of distractions when using KineActiv	3.0	0.47
13. Using KineActiv was boring for me	1.6	0.52
14. KineActiv was fun for me to use	4.4	0.52
15. I felt that I had the control over my rehabilitation process with KineActiv	3.9	0.74
16. I was frustrated with what I was doing when using KineActiv	1.4	0.52
